# Real-world data on T-DM1 efficacy – results of a single-center retrospective study of HER2-positive breast cancer patients

**DOI:** 10.1038/s41598-019-49251-5

**Published:** 2019-09-04

**Authors:** Max Hardy-Werbin, Vanesa Quiroga, Beatriz Cirauqui, Margarita Romeo, Eudald Felip, Iris Teruel, Juan Jose Garcia, Carlos Erasun, Sofia España, Marc Cucurull, Elisabeth Montprade, Juan Carlos Pardo, Dania Carballo, Jose Maria Velarde, Mireia Margeli

**Affiliations:** 1 Cancer Research Program, IMIM (Hospital del Mar Medical Research Institute), Barcelona, Spain; 2 B-ARGO Group, Institut Català d’Oncologia, Medical Oncology Service, Hospital Germans Trias i Pujol, Badalona, Spain; 3 Escuela de Medicina y Ciencias de la Salud, Tecnológico de Monterrey, Mexico; 4 Statistical Department, Institut d’Investigació en Ciències de la Salut Germans Trias i Pujol (IGTP), Medical Oncology Service, Hospital Germans Trias i Pujol, Badalona, Spain

**Keywords:** Drug discovery, Chemistry

## Abstract

T-DM1 is an antibody drug conjugate that combines trastuzumab with emtansine via a stable thioether linker. In two phase III clinical trials, EMILIA and TH3RESA, T-DM1 was shown to be effective in HER2-positive metastatic breast cancer patients who had progressed to taxanes and trastuzumab. We have performed a real-world study to complement the findings of the clinical trials. From 2012 to 2016, 15 patients with HER2-positive breast cancer who had progressed to prior treatment received T-DM1 at our center. We have retrospectively analyzed outcomes in these patients and compared our findings with those of the two clinical trials. Progression-free survival (PFS) was 10 months compared with the 9.6 months of the EMILIA trial and the 6.2 months of the TH3RESA trial, overall survival was 34 months compared with the 29.9 months of the EMILIA trial and the 22.7 months of the TH3RESA trial. PFS was ≥12 months in five patients, three of whom attained a PFS of ≥23 months. Among five patients with metastases of the central nervous system, PFS was six months, OS was not reached, and the objective response rate was 80%. Our findings are in line with those of the EMILIA study and slightly superior to those of the TH3RESA study. In our series of patients, T-DM1 has demonstrated efficacy in the treatment of HER2-positive metastatic breast cancer. Our real-world data thus confirm and support the findings of the two major phase III trials and indicate the usefulness of T-DM1 in routine clinical practice.

## Introduction

Breast cancer is the most common malignancy in women worldwide and is the cause of more than 570,000 deaths every year^1,2^. Over the last ten years, a new molecular classification of breast cancer has been developed that includes the human epidermal growth receptor 2 (HER2), a member of the ErbB family. HER2 is amplified in about 20% of invasive breast cancers and is associated with a more aggressive disease and poor clinical outcome^3,4^. Treatment of patients with HER2-positive breast cancer has evolved rapidly since the identification of HER2 as an ideal target for anticancer agents. The combination of trastuzumab, pertuzumab, and a taxane significantly prolonged progression-free survival (PFS) in a randomized trial^5^ and is now the standard first-line treatment for HER2-positive breast cancer. However, patients will eventually relapse and require a second line of treatment. Several studies have shown that the combination of trastuzumab plus emtansine (T-DM1) is active in HER2-positive breast cancer patients who have progressed after prior treatment^6–9^. Recent data from KATHERINE trial, among patients with HER2-positive early breast cancer who had residual invasive disease after completion of neoadjuvant therapy, has shown a reduction of 50% of the risk of recurrence of invasive breast cancer or death with adjuvant T-DM1 instead of trastuzumab alone^10^.

T-DM1 is an antibody drug conjugate that combines the action of trastuzumab with that of emtansine. Trastuzumab is a humanized monoclonal antibody that binds selectively to the HER2 membrane receptor, inducing apoptosis and activating cell immunity. Emtansine is an antimicrotubule that inhibits tubulin polymerization. *In vitro* studies have shown that emtansine is 40 times more powerful than taxanes, maintaining efficacy without increasing toxicity. In T-DM1, trastuzumab and emtansine are linked by a stable thioether linker that stabilizes the conjugate until it reaches the target HER2 receptor, where it undergoes receptor-mediated internalization and subsequent lysosomal degradation. Finally, emtansine is released into the cytoplasm, where it acts to disrupt microtubules and inhibit HER2 signaling, resulting in cell-cycle arrest and apoptosis^11,12^.

The randomized phase III EMILIA trial included 991 HER2-positive metastatic or locally advanced breast cancer patients who had previously received trastuzumab and a taxane. Patients were randomized to receive T-DM1 (3.6 mg/kg every 21 days) or capecitabine plus lapatinib. Results showed a clear advantage for T-DM1 over capecitabine plus lapatinib in PFS (9.6 vs 6.4 months; hazard ratio [HR], 0.65; p < 0.001) and overall survival (OS) (30.9 vs 25.1 months; HR, 0.68; p < 0.001). The T-DM1 arm also showed a higher response rate (43.6% vs. 30.8%; p < 0.001) and fewer grade 3–4 adverse events (41% vs 57%)^6,7^. Based on the results of the EMILIA trial, in February 2013, T-DM1 was approved by the US Food & Drug Administration (FDA) for the second-line treatment of HER2-positive breast cancer^13^.

In another randomized phase III trial, TH3RESA, 602 patients with metastatic HER2-positive breast cancer were randomized to receive either T-DM1 or a treatment of the physician’s choice. All patients had progressed to at least two previous anti-HER regimens. Patients in the T-DM1 arm had longer PFS (6.2 vs 3.3 months; HR, 0.52; p < 0.001) and OS (22.7 vs 15.8 months; HR, 0.68; p < 0.001)^8,14^.

Based on this clear evidence of clinical benefit, T-DM1 is now the standard treatment for patients with HER2-positive breast cancer who progress during or within 12 months after adjuvant treatment with trastuzumab and for those who relapse after initially responding to treatment with trastuzumab plus a taxane, with or without pertuzumab.

Although clinical trials are the accepted standard for establishing the efficacy of a treatment regimen, they evaluate a standardized therapy in a selected group of patients and thus may fail to assess complex interactions involved in the delivery of care in routine clinical practice. A study of real-world data can fill in these gaps and can generate long-term efficacy data to complement findings of clinical trials^15^. In the present retrospective real-world study, we have evaluated the efficacy of T-DM1 in a series of HER2-positive breast cancer patients treated in our center.

## Methods

From August 2012 to May 2016, 15 patients with HER2-positive breast cancer were treated with T-DM1 at Hospital Germans Trias i Pujol, Badalona, Spain. We retrospectively collected clinical characteristics and data on PFS, OS, response and toxicity from hospital records on these patients. The study was approved by the hospital ethics committee (“CEIC DEL HOSPITAL UNIVERSITARIO GERMANS TRIAS I PUJOL”) and all patients gave their signed informed consent.

PFS was calculated from the beginning of treatment with T-DM1 until disease progression or death from any cause. OS was calculated from the beginning of treatment with T-DM1 until death from any cause. Response was evaluated in accordance with Response Evaluation Criteria In Solid Tumors (RECIST guideline version 1.1). Toxicity was evaluated by NCI CTCAE (version 4.0). Kaplan Meier curves with their 95% confidence intervals (CIs) were drawn for PFS and OS and compared with a log-rank test. Significance was set at p ≤ 0.05. All analyses were performed with SPSS v24.

### Ethical approval

All procedures performed in studies involving human participants were in accordance with the ethical standards of the institutional and/or national research committee and with the 1964 Helsinki declaration and its later amendments or comparable ethical standards. The study was approved by the hospital ethics committee (“CEIC del HOSPITAL UNIVERSITARIO GERMANS TRIAS I PUJOL”) and all patients gave their signed informed consent.

## Results

### Patients

Table 1 displays the characteristics of the patients. Median age was 48 years, and 53.4% were pre-menopausal. The ECOG status was 0 in 20% of patients, 1 in 73.3% of patients, and 2 in 6.7% of patients at time of the therapy. The majority (73.3%) of patients were triple-positive. *De novo* metastatic disease was diagnosed in 26.6%, whereas the remaining 73.4% of patients were diagnosed with locally or locally advanced disease and later became metastatic. Metastases with visceral disease involvement were present in 60% of the patients, five (33.3%) had metastases in the central nervous system (CNS). The median lines of previous therapy was 2.5, all patients had previously received trastuzumab either as an adjuvant treatment or for metastatic disease, 80% of patients had received capecitabine with lapatinib for advanced disease, 60% of patients had received anthracyclines (9 patients received them as adjuvant or neoadjuvant therapy, and 2 patients received liposomal anthracyclines for metastatic setting), 86.7% of patients received taxanes (9 patients received them as adjuvant or neoadjuvant therapy, and 7 patients for metastatic setting), 47% of patients received hormonal therapy (6 patients received them as adjuvant or neoadjuvant therapy, and seven patients for metastatic setting), 1 patients was treated with Carboplatin within the scheme of docetaxel, carboplatin trastuzumab in the adjuvant setting, 2 patients were treated with the scheme CMF in the adjuvant setting. Table 2 displays all the previous therapies for the adjuvant/neoadjuvant and metastatic setting. The majority (66.6%) of patients received T-DM1 as the third or fourth line of treatment for metastatic disease, and the median number of cycles administered was 9 (1–42).

**Table 1:** Characteristics of 15 HER2-positive breast cancer patients.

Characteristic	N = 15
	N (%)
**Age**, **yrs**	
Median (range)	48 (36–71)
Pre-menopausal	8 (53.4)
Post-menopausal	7 (46.6)
**ECOG performance status**	
0	3 (20)
1	11 (73.3)
2	1 (6.7)
**Disease extent at diagnosis**	
Local	5 (33.3)
Locally advanced	6 (40)
Metastatic	4 (26.7)
HER2-positive	15 (100)
**Hormone receptor status**	
ER+ and/or PR+	11 (73.3)
ER− and PR−	4 (26.7)
**Visceral disease involvement**	
Yes	9 (60)
No	6 (40)
**Number of metastatic sites**	
<3	9 (60)
≥3	6 (40)
**CNS metastases**	
Yes	5 (33.3)
No	10 (66.7)
**Previous treatment**	
Number of treatment lines – Median (range)	2.5 (1–7)
Hormonal therapy	7 (47%)
Trastuzumab	15 (100)
Capecitabine-lapatinib	12 (80)
Anthracyclines	9 (60)
Taxanes	13 (86.7)
**T-DM1 treatment line**	
Second	3 (20)
Third	5 (33.3)
Fourth	5 (33.3)
Fifth or more	2 (13.4)
Number of T-DM1 cycles – Median (range)	9 (1–42)

ECOG, Eastern Cooperative Oncology Group; ER, estrogen receptor; PR, progesterone receptor; CNS, central nervous system.

**Table 2:** Previous therapies.

Characteristic	N = 15
	N (%)
**Previous treatment for advanced disease**	
Number of treatment lines – Median (range)	2.5 (1–7)
Hormonal therapy	6 (40)
Trastuzumab	15 (100)
Capecitabine-lapatinib	12 (80)
Lyposomal Anthracyclines	2 (13.4)
Taxanes	7 (47)
**Previous treatment for adjuvant/neoadjuvant therapy**	
Hormonal therapy	7 (47)
Trastuzumab	14 (94)
Anthracyclines	9 (60)
Taxanes	9 (60)
CMF	2 (13.4)
Carboplatin	1 (6.7)

CMF, Cyclophosphamide, Methotrexate, Fluorouracil.

### Clinical outcomes

**Figure 1:**
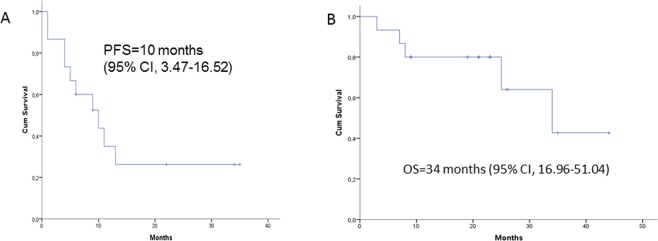
(**a**) Progression-free survival and (**b**) overall survival to T-DM1 in 15 HER-positive breast cancer patients.

All 15 patients were evaluable for PFS, OS, and response. Median PFS was 10 months (95% CI, 3.47–16.52) (Fig. 1A). Median OS was 34 months (95% CI, 16.96–51.04) (Fig. 1B). The objective response rate was 73.3%. Four patients (26.6%) achieved a complete response, seven (46.7%) a partial response, one (6.7%) had stable disease, and three (20%) progressed (Table 3).

**Table 3:** Response to T-DM1 in 15 HER2-positive breast cancer patients.

Response	N (%)
Complete response	4 (26.7%)
Partial response	7 (46.6%)
Stable disease	1 (6.7%)
Progressive disease	3 (20%)

**Figure 2:**
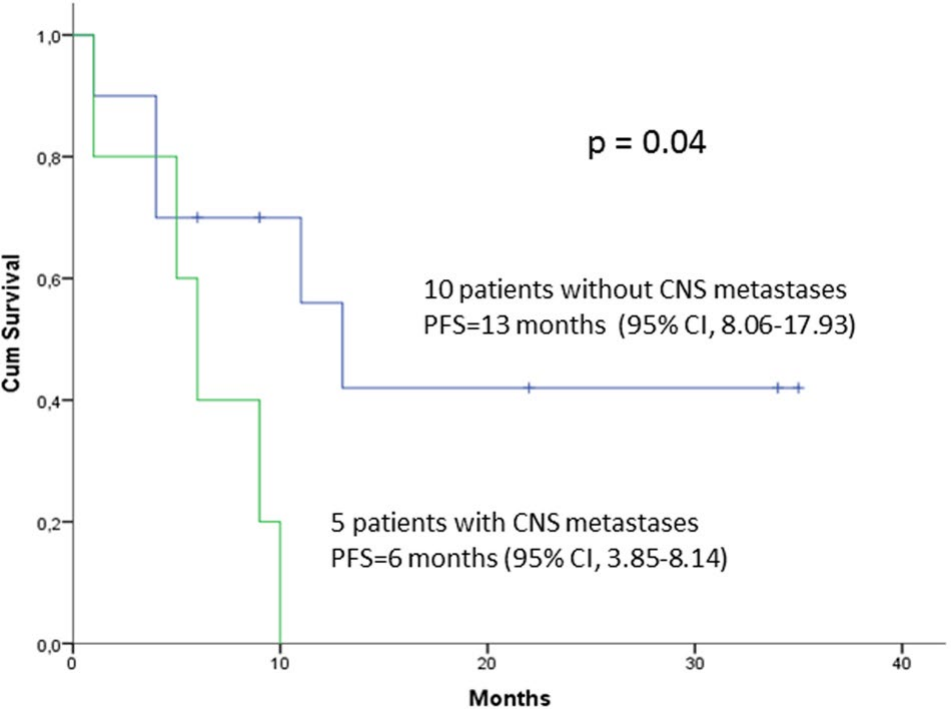
Progression-free survival in ten patients without metastases of the central nervous system (CNS) (blue line) and in five patients with CNS metastases (green line).

Among the five patients with CNS metastases, PFS was six months (95% CI, 3.85–8.14), compared to 13 months (95% CI, 8.06–17.93) for the ten patients without CNS metastases (p = 0.04) (Fig. 2). OS was not reached in patients with CNS metastases. The objective response rate for patients with CNS metastases was 80%, with one complete response, three partial responses, and one progressive disease.

The main grade 3–4 toxicities were elevated transaminases (6.7%), thrombocytopenia (6.7%), anemia (6.7%), and neutropenia (6.7%) (Table 4). In the one patient with grade 3 neutropenia, it was a pre-existing condition.

**Table 4:** Main adverse events.

Adverse Event	Any Grade	Grade 3–4
ElevatedTransaminases	11 (73.3%)	1 (6.7%)
Thrombocytopenia	8 (53.4%)	1 (6.7%)
Anemia	7 (46.6%)	1 (6.7%)
Neutropenia	7 (46.6%)	1 (6.7%)
Hypokalemia	7 (46.6%)	1 (6.7%)

## Discussion

On the basis of the results of two clinical trials, EMILIA^6,7,9^ and TH3RESA^8,13^, T-DM1 has become accepted as the standard of treatment for HER2-positive breast cancer, both as second- or further-line treatment for advanced or metastatic disease and as first-line treatment in the case of early progression to adjuvant treatment. The present retrospective study of a small series of patients treated in a single center provides real-world data to complement and support the findings of the two large clinical trials (Table 5).

**Table 5:** Patient characteristics and outcomes in the present study, the EMILIA study, and the TH3RESA study.

	Present Study N = 15 N (%)	EMILIA (Verma *et al*. 2012) N = 991 N (%)		TH3RESA (Krop *et al*. 2017; Krop *et al*. 2014) N = 602 N (%)	
Treatment	T-DM1	Cap + Lap N = 496	T-DM1 N = 495	PC N = 198	T-DM1 N = 404
Age, median (range)	45 (36–71)	53 (24–83)	53 (25–84)	54 (28–85)	53 (27–89)
**ECOG PS**					
0	3 (20)	312 (64)	299 (61)	82 (41)	180 (45)
1	1 (73)	176 (36)	194 (39)	101 (51)	200 (50)
2	1 (7)	0	0	15 (8)	22 (5)
**HR status**					
ER+ and/or PR+	11 (73)	263 (62)	282 (57)	103 (52)	208 (51)
ER− and PR−	4 (27)	224 (45)	202 (41)	85 (43)	185 (46)
Unknown		9 (2)	11 (2)	10 (5)	11 (3)
**Visceral disease involvement**					
Yes	9 (60)	335 (68)	334 (67)	150 (76)	302 (75)
No	6 (40)	161 (32)	161 (33)	48 (24)	102 (25)
**Number of metastatic sites**					
<3	9 (60)	307 (62)	298 (60)	na	na
≥3	6 (40)	175 (35)	189 (38)		
Unknown	0	14 (3)	8 (2)		
CNS metastases	5 (33)	50 (10)	45 (9)	27 (14)	40 (10)
**Previous treatment**					
Median number of treatment lines	2.5	3	3	4	4
Taxanes	13 (87)	494 (100)	493 (100)	na	na
Anthracyclines	9 (60)	302 (61)	303 (61)	na	na
Hormonal therapy	7 (47)	204 (41)	205 (41)	na	na
Trastuzumab	15 (100)	495 (100)	205 (100)	198 (100)	404 (100)
Lapatinib	12 (80)	0	0	198 (100)	404 (100)
PFS (months)	10	6.4	9.6	3.3	6.2
OS (months)	34	25.1	30.9	15.8	22.7
ORR	73.3%	30.8%	43.6%	9%	31%
**Grade 3–4 toxicities**					
Elevated transaminases	1 (6.7)	10 (2.2)	35 (7.2)	4 (2)	24 (6)
Thrombocytopenia	1 (6.7)	1 (0.2)	64 (12.9)	3 (1)	19 (4)
Anemia	1 (6.7)	8 (1.6)	13 (2.7)	5 (3)	11 (3)
Neutropenia	1 (6.7)	21 (4.3)	10 (2.0)	29 (16)	10 (3)
Hypokalemia	1 (6.7)	20 (4.1)	11 (2.2)	na	na

Cap + Lap, capecitabine plus lapatinib; PC, physician’s choice; ECOG, Eastern Cooperative Oncology Group; PS, performance status; HR, hormone receptor; ER, estrogen receptor; PR, progesterone receptor; na, data not available; CNS, central nervous system; PFS, progression-free survival; OS, overall survival; ORR, overall response rate.

In our HER2-positive breast cancer patients treated with T-DM1, PFS was 10 months, which is similar to the 9.6 months observed in the EMILIA study and longer than the 6.2 months observed in the TH3RESA study, which included patients who had received a greater number of prior treatment lines. Similarly, OS in our patients (34 months) was somewhat longer than that attained by the patients in the EMILIA study (30.9 months) and the TH3RESA study (22.7 months). In fact, PFS was longer than 12 months in five of our patients, including three who were progression-free for more than 23 months. None of these patients had visceral metastatic disease. Other cases of exceptional and durable responses to T-DM1 have been described in literature, in special in patients without visceral disease^16^.

In fact, the slightly lower overall rate of metastases in our patients may have been a factor in the longer PFS and OS observed in our series of patients as described in other series^17^. Lung, liver, and/or CNS metastases were present in 60% of our patients, 67% of the EMILIA patients, and 75% of the TH3RESA patients receiving T-DM1. Moreover, 46% of our patients had metastases in three or more sites, compared to 37% in the EMILIA study and 64% in the TH3RESA study. In contrast with the clinical trials, we included all patients who could have benefited from T-DM1. The inclusion of these patients with a better prognosis, which enabled them to receive further lines of treatment, may have introduced a bias in our study that was not present in the clinical trials.

Nonetheless, five (33.3%) of our patients had CNS metastases, compared to 9% and 10% in the EMILIA and TH3RESA trials, respectively. A sub-analysis of patients with CNS metastases in the EMILIA study found no differences in PFS between patients treated with T-DM1 and those treated with capecitabine and lapatinib (5.9 vs 5.7 months). However, in patients with treated, asymptomatic CNS metastases at baseline, T-DM1 was associated with significantly longer OS^7^. All of our five patients with CNS metastases had previously received local treatment and were administered T-DM1 at progression either in the CNS or another site. PFS for these patients was six months, which is along the lines of the EMILIA study, while OS was not reached in this subgroup of patients. Interestingly, a small study of T-DM1 in HER2-positive breast cancer patients with brain metastases found that T-DM1 was active against the brain metastases as well^18,19^.

Eleven of our 15 patients (73.3%) were triple-positive, compared to 55% in the EMILIA study and 52% in the TH3RESA study. The higher proportion of triple-positive patients in our series may be a result of the FDA approval of T-DM1, which paved the way for its administration in patients who had already received multiple lines of treatment. We can speculate that these patients may have already attained longer survival in previous treatment lines, which allowed them to receive T-DM1 in more advanced treatment lines. This may have introduced a certain bias into the selection of our patients which would not have been present in the clinical trials. This could be a reflection of the period in which we treated these patients within the expanded access program of T-DM1 (2012–2016), as 80% of these patients were assessed in the second line or further, hence HER2-positive but hormone-receptor negative patients with poorer prognosis were deemed not suitable due to aggressive disease behavior. This could have impacted as a negative bias in selection. Although triple-positive breast cancer patients generally attain lower rates of pathological complete response to neoadjuvant treatment^20–22^, our findings suggest that this poorer initial response does not carry over in the long term to treatment with T-DM1. The median age of our patients was 48 years, compared to 53 in the EMILIA study, while 83% of the patients in the TH3RESA study were younger than 65.

The majority of our patients had already received two or more previous lines of treatment, including hormonotherapy plus trastuzumab. The median number of prior treatment lines in our patients was 2.5, compared to four and three in the TH3RESA and EMILIA studies, respectively. However, five of our patients were treated as a fourth or further line of treatment, and one patient received T-DM1 as the eighth line of treatment. All of the patients in the TH3RESA study had received at least two lines of previous treatment, including trastuzumab, lapatinib, or taxanes. In the EMILIA study, the majority of patients (88.9% in the T-DM1 group and 88.3% in the capecitabine plus lapatinib group) had already received prior systemic treatment in the metastatic setting. The remaining 12.1% of patients in the T-DM1 group and 11.7% of patients in the capecitabine plus lapatinib group had received prior systemic therapy in the early stage setting. Our patients had received prior trastuzumab, capecitabine and lapatinib, anthracyclines, and/or taxanes; but the median number of previous lines was slightly lower than that of the TH3RESA study and clearly lower than that of the EMILIA study, which may explain the longer PFS and OS attained by our patients.

In the present study, we have observed a higher rate of elevated transaminases and neutropenia than that reported for the EMILIA and TH3RESA studies. A safety analysis of six studies of T-DM1 in HER2-positive breast cancer^23^, including 884 patients, found that that the most frequent adverse effect was asthenia, followed by nausea and vomiting, while 32% of patients had thrombocytopenia and 23% had elevated transaminases. Overall, our patients had higher rates of hepatotoxicity and hematological toxicities, although most cases were grade 1–2. In fact, only one patient had grade 3 neutropenia, and this was a pre-existing condition. These higher rates of grade 1–2 toxicity in our study may be due to a less strict control of the T-DM1 dose than would be necessary in a clinical trial or to a greater number of T-DM1 cycles. Although the median of nine cycles of T-DM1 in our series was in line with the 10.1 and 10.5 cycles in the EMILIA and TH3RESA studies, in fact, five of our patients received more than 12 cycles.

In conclusion, in our small series of patients, T-DM1 has demonstrated efficacy in the treatment of HER2-positive metastatic breast cancer, with an excellent safety and tolerability profile. Our real-world data thus confirm and support the findings of the two major phase III trials and indicate the usefulness of T-DM1 in routine clinical practice.
